# An Assessment of the Distribution and Prevalence of Benign Intraoral Pathologies

**DOI:** 10.3390/diagnostics15030350

**Published:** 2025-02-03

**Authors:** Sinan Yasin Ertem, Furkan Uz

**Affiliations:** School of Dentistry, Ankara Yildirim Beyazit University, Ankara 06010, Turkey; furkanuz@aybu.edu.tr

**Keywords:** oral, pathology, intraoral, benign, prevalence

## Abstract

**Objectives:** The aim of this study is to evaluate the histopathological examinations of biopsy samples obtained from patients, and to determine the prevalence, age, and gender distribution of intraoral benign lesions. The study examines the distribution of all benign intraoral pathologies, including both soft tissue and hard tissue lesions, through which the goal is to contribute to clinical diagnostic processes. **Methods:** The histopathological evaluation results of 235 patients who underwent biopsy between 2021 and 2024 were retrospectively analyzed. Out of 235 patients, 123 (52.34%) were female and 112 (47.66%) were male. Lesions were categorized into two groups: soft tissue lesions and intraosseous lesions. The frequency, gender distribution, and age ranges of these lesions were assessed. Lesions exhibiting dysplasia or malignancy were excluded from the study. **Results:** The most common benign intraoral lesion was identified as the radicular cyst, observed in 69 patients. The age range for radicular cysts varied from 8 to 80 years, with 30 cases in females and 39 in males. The most frequently encountered soft tissue lesion was traumatic fibroma, which constituted 25.33% (19 patients) of all soft tissue lesions. Traumatic fibromas were observed in patients aged between 12 and 62 years. In terms of overall prevalence among all benign intraoral pathological lesions, radicular cysts ranked first (29.36%), followed by periapical granulomas (15.31%), dentigerous cysts (11.06%), and traumatic fibromas (8.08%). The occurrence of soft tissue lesions was significantly higher in females (66.66%) compared to males (33.34%). **Conclusions:** There are no recent studies in the literature evaluating the prevalence and demographic distribution of intraoral benign lesions. The most common lesions diagnosed in the study are typically associated with inflammation and irritation. The most common hard tissue lesion was the radicular cyst, which was seen across a wide age range and in similar proportions in men and women. Among soft tissue lesions, traumatic fibroma was the most common, particularly in women, and was seen across a wide age range. In terms of gender distribution, soft tissue lesions were twice as common in women as in men.

## 1. Introduction

Intraoral pathologies can develop from various tissues within the oral cavity, including the epithelium, connective tissue, muscle, bone, and salivary glands. The development of a wide range of pathologies in the oral and maxillofacial region is primarily attributed to genetic factors and exposure to harmful environmental agents. While histopathological analysis is essential to diagnose and accurately characterize the lesion and create an appropriate treatment plan based on its characteristics, understanding the prevalence of these lesions is crucial for preliminary diagnosis. This knowledge aids in determining clinical approaches and enhancing physician awareness [1]. Indications for intraoral biopsy include oral lesions that persist for more than 14 days without healing, lesions such as leukoplakia or erythroplakia, firm palpable nodules or masses, lesions exhibiting color changes, abnormal tissue alterations due to chronic irritation or trauma, swelling or pain in the salivary glands, or lesions suspected to be malignant tumors.

The literature includes various studies on intraoral lesions analyzing prevalence; however, these studies predominantly focus on specific subgroups or categorize lesions based on parameters such as age, gender, or etiology. Only a few studies have evaluated intraoral benign pathologies without subgrouping, etiological factors, or age restrictions. For instance, Ono et al. [2] conducted a study in 2002 that analyzed the distribution of intraoral benign pathologies. However, since the data in that study were collected between 1972 and 1998, there remains a significant lack of contemporary data regarding the distribution and frequency of intraoral benign lesions. A review of the English-language literature from the past five years reveals no studies that evaluate intraoral benign pathologies without restrictions on subgroups or age.

Benign lesions constitute a significant proportion of the pathologies observed in the oral region. With advancements in technology, changing dietary patterns, and the increasing prevalence of harmful habits, the factors that impact the oral environment are continuously evolving. Consequently, understanding the current distribution of benign intraoral lesions is crucial. Providing updated data is essential to facilitate early diagnosis, enhance awareness, and inform clinical practice.

This retrospective study aims to address this gap by analyzing the current prevalence and demographic distribution of intraoral benign lesions based on biopsy specimens from 235 patients referred for histopathologic analysis. Additionally, the study seeks to enhance clinical predictability in diagnosing intraoral pathologic lesions and to support the efficient utilization of healthcare resources.

## 2. Materials and Methods

### 2.1. Data Collection

This study was conducted through an analysis of the histopathological results of 235 patients who consecutively applied to the Department of Oral and Maxillofacial Surgery at Ankara Yildirim Beyazit University, Faculty of Dentistry, between 2021 and 2024, and underwent biopsy following clinical examination. All biopsy specimens were sent to the Gazi University Faculty of Dentistry, Department of Oral Pathology, for examination. Histopathological analysis was primarily performed on paraffin-embedded sections stained with hematoxylin and eosin. When necessary, immunohistochemical staining was utilized to confirm specific diagnoses.

### 2.2. Study Design

A total of 123 female and 112 male patients were included in this study. For soft tissue lesions suspected to be pathological based on intraoral examination, patients were instructed to follow oral hygiene protocols and use chlorhexidine mouthwash for two weeks. Lesions that showed no regression were biopsied using either incisional or excisional techniques, depending on lesion size. Incisional biopsies were performed on ulcerative, white, or red lesions with clinical suspicion of malignancy, while excisional biopsies were conducted for other soft tissue lesions and hard tissue lesions. Specimens were preserved in 10% formalin solution and sent to the histopathology laboratory on the same day for examination. Only lesions with a definitive diagnosis following histopathological evaluation were included in the study.

### 2.3. Data Analysis

The collected data were recorded in Microsoft Excel, which categorized each case by gender, age, and histopathological diagnosis. The data for patients with soft tissue lesions were subdivided into 12 diagnostic groups based on concordance between clinical and pathological diagnoses, and are presented in Table 1. The prevalence of each soft tissue lesion was calculated as a percentage of the total number of soft tissue lesions. Intraosseous lesions were classified into nine subgroups, with the data summarized in Table 2. The prevalence of each intraosseous lesion type was calculated as a percentage of the total number of intraosseous lesions. For each lesion type, the mean age, age range, number of male and female patients, and male-to-female ratio were also calculated and included in the tables.

**Figure 1:**
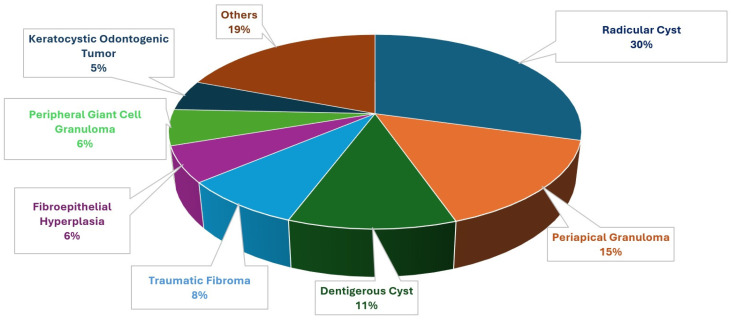
Frequency rates of all intraoral benign pathologies without subgrouping.

**Figure 2:**
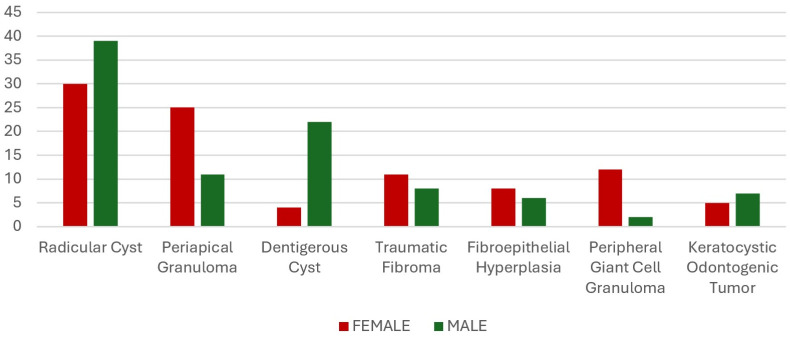
Female and male comparison of the most common intraoral pathologies.

Additionally, the prevalence of all benign intraoral pathologies was assessed collectively, and categorized into eight groups: radicular cysts, periapical granulomas, dentigerous cysts, traumatic fibromas, fibroepithelial hyperplasia, peripheral giant cell granulomas, keratocystic odontogenic tumors, and other lesions, as illustrated in Figure 1. The number of male and female patients for the seven most prevalent lesion types is presented separately in Figure 2.

### 2.4. Inclusion and Exclusion Criteria

The inclusion criteria for this study were as follows: the presence of a pathological lesion in either soft or hard tissue; treatment at the Department of Oral and Maxillofacial Surgery, Ankara Yildirim Beyazit University; a biopsy performed; and a definitive diagnosis established through histopathological examination. Patients were included without any demographic limitations. The exclusion criteria included missing pathology reports, incomplete demographic information, insufficient clinical details, inconclusive biopsy results, and histopathological findings indicative of dysplasia or malignancy. Furthermore, premalignant lesions and pathologies that demonstrated cellular-level dysplasia were excluded from the study.

### 2.5. Ethical Considerations

Ethical approval was obtained from the Health Sciences Ethics Committee of Ankara Yildirim Beyazit University in October 2024 (Approval Code: 07/865).

## 3. Results

Among the patients with histopathologically confirmed intraoral benign lesions, 52.34% were female (123 patients) and 47.66% were male (112 patients). Intraosseous lesions were identified in 160 patients (68.08%), while 75 patients (31.92%) had soft tissue lesions. Intraosseous lesions were found in 87 male patients (54.37%) and 73 female patients (45.63%). Soft tissue lesions were observed in 50 female patients (66.66%) and 25 male patients (33.34%). Histopathological evaluation of soft tissue lesions revealed that the most common soft tissue lesion in the intraoral region was traumatic fibroma, which was detected in 19 patients (25.33%). Among patients with traumatic fibroma, 57.89% were female and 42.11% were male, with an age range of 12 to 62 years. Fibroepithelial hyperplasia and peripheral giant cell granuloma were the second most common soft tissue lesions, with each one found in 14 patients (18.66%). The average age of patients with fibroepithelial hyperplasia was 45.92 years, with an age range of 14 to 81 years. The mean age for peripheral giant cell granuloma was 49.53 years, with an age range of 8 to 75 years. Rare soft tissue lesions including amalgam tattoo, lipoma, verruca vulgaris, and lupus erythematosus were observed in only one patient each (1.33%) (Table 1).

In the analysis of intraosseous lesions, the most frequently observed pathology was a radicular cyst, detected in 69 patients, which comprised 43.12% of the patient group. Among the patients with radicular cysts, 30 were female and 39 were male. The age range for radicular cysts was from 8 to 80 years, with an average age of 43.33 years. The second most common lesion was periapical granuloma, observed in 36 patients (22.50%), with a mean age of 41.05 years. Dentigerous cysts were the third most common lesion, found in 26 patients (16.25%), whose average age was 31.84 years. A keratocystic odontogenic tumor (KOT) was observed in 12 patients (7.50%). In terms of gender distribution, radicular cysts were more frequent in males (56.53%). Periapical granulomas were more common in females, 25 cases of which were in women and 11 in men. Dentigerous cysts were more prevalent in males (84.62%). Condensing osteitis was observed in two patients (1.25%), while periapical cemental dysplasia and a residual cyst were found in one patient each (0.62%) (Table 2).

When soft and hard tissue lesions were considered collectively, the most frequently observed intraoral benign pathological lesion was radicular cysts (29.36%, 69 patients), followed by periapical granuloma (15.31%, 36 patients), dentigerous cyst (11.06%, 26 patients), traumatic fibroma (8.08%, 19 patients), fibroepithelial hyperplasia (5.95%, 14 patients), peripheral giant cell granuloma (5.95%, 14 patients), and keratocystic odontogenic tumor (5.10%, 12 patients) (Figure 1).

**Figure 3:**
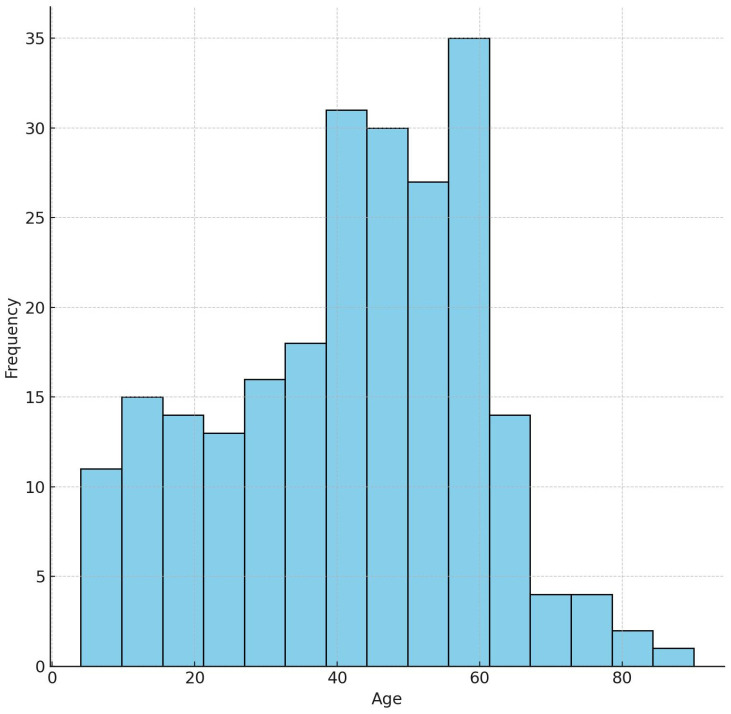
The overall distribution of ages in patients.

An analysis of the age distribution of all patients revealed that 48.51% (114 patients) were between 40 and 60 years of age, although intraoral pathologic lesions demonstrated a broad age range (Figure 3). The mean age of the patients with soft tissue lesions that required histopathological examination was 46.04 years. The mean age of patients with intraosseous lesions was 39.48 years. The overall average age of all patients that underwent histopathological evaluation was calculated as 41.57 years.

## 4. Discussion

Knowledge of the prevalence and demographic characteristics of oral pathologies is very important for accurate diagnosis of lesions and treatment planning. The existing literature on this subject primarily focuses on an analysis of specific groups, with no current studies addressing the investigation of intraoral benign pathologies. Previous studies on this subject have predominantly focused on specific age groups such as children, adolescents, and young to middle-aged adults [3,4,5,6,7,8,9,10,11,12]. While some studies in the literature address either soft or hard tissue pathologies exclusively, others categorize pathological lesions into distinct subgroups [13,14,15,16]. In the present study, the distribution of all benign intraoral lesions was analyzed without restrictions on age groups or pathology subgroups, aiming to provide a comprehensive understanding of their prevalence in clinical practice.

Although the age distribution of patients with oral pathologies encompasses a wide range, it is generally accepted that these conditions are most prevalent during the third, fourth, and fifth decades of life [17,18]. Similarly, the mean age of the patients in this study was calculated to be 41.57 years, where 133 of the 235 patients fall between the ages of 30 and 59. These findings indicate that individuals aged 30–59 years constitute a high-risk group for oral pathologies.

Previous studies in the literature have demonstrated variability in the gender distribution of oral and maxillofacial pathologies. Al Hindi et al. [1] reported a prevalence of 52.8% for oral pathology in females, and similar findings have been consistently observed, indicating a higher occurrence of oral pathological lesions in women [19,20,21]. This trend has been attributed to greater health awareness and more frequent visits to healthcare providers among women [1]. Consistent with these observations, the present study also found a higher prevalence in females (52.34%) compared to males (47.66%). Furthermore, of the patients with soft tissue lesions, 50 were female (66.66%) and 25 were male (33.34%), with soft tissue lesions occurring twice as frequently in females as in males.

Several studies have reported that soft tissue lesions are more frequently observed among oral pathologies [22]. This is often attributed to the fact that clinicians tend to manage extraosseous oral lesions independently and without referral, whereas intraosseous lesions, which typically require more advanced surgical interventions, are more frequently referred to oral and maxillofacial surgery. However, in this study, the number of soft tissue lesions was 75 (31.91%), while the number of intraosseous lesions was 160 (68.09%). The higher proportion of intraosseous oral lesions compared to soft tissue lesions is likely due to the specific patient group treated in this study, which was managed in an oral and maxillofacial surgery clinic. It is widely recognized in clinical practice that all lesions suspected to be pathological should undergo histological examination. However, soft tissue lesions sometimes do not require histopathological analysis, as malignancy can often be ruled out through clinical evaluation, treatment, and follow-up, in contrast to intraosseous lesions. This may explain the higher incidence of intraosseous lesions observed in this study.

In this study, the highest prevalence rates were observed in lesions resulting from inflammation and reactive processes. Among intraosseous lesions, radicular cysts were the most common, accounting for 43.12%. Consistent with the literature, inflammatory cysts are the most prevalent pathological findings, both overall and particularly among intraosseous lesions. It was anticipated that these lesions, which are known to arise due to inflammation, would be found at a high rate in this study. Several studies in the literature have reported cystic lesions as the second most common pathology, with radicular cysts being the most frequently occurring odontogenic cysts [23].

The prevalence of pathologies in the oral region exhibits substantial heterogeneity. Several studies in the literature report that reactive lesions are more commonly observed [24,25]. Traumatic fibromas, which are known to develop due to chronic irritation, are the most frequent pathological findings among soft tissue lesions. In a study by Sangle et al. [26], traumatic fibromas were reported as the most common pathologies in the third and fourth decades of life, with a prevalence of 37.4%. Similarly, in this study, traumatic fibromas were the most common soft tissue lesion, occurring in 25.33% of cases (19 patients), with a mean age of 47.44 years. Fibroepithelial hyperplasia and peripheral giant cell granulomas, both of which are known to arise from reactive processes due to irritation, were the next most common lesions after traumatic fibromas.

A limitation in the evaluation of prevalence findings in this study, particularly for common benign pathologies, is that they can often be diagnosed without the need for histopathological examination. Consequently, the results may vary depending on differences in referral procedures or the clinicians’ level of experience. In the literature, periapical granulomas have been reported as the most common pathological lesions in some studies [27], while other studies have ranked them as the second most common lesions [1]. In this study, periapical granulomas, categorized as intraosseous lesions, were found to be the second most common lesion. This may be attributed to the fact that periapical granulomas do not typically require histopathological examination. Clinically, granulomas are known to be the most prevalent lesions. Similarly, Jones et al. reported that periapical granulomas are the most common lesions among all pathologies [19].

In this study, only one patient with periapical cemental dysplasia and two patients with condensing osteitis were identified upon histopathologic examination. Based on clinical experience, condensing osteitis typically only requires follow-up care and is less frequently detected in histopathologic examinations due to its rare need for surgical intervention. The asymptomatic nature of condensing osteitis makes it difficult to diagnose clinically, and it is usually detected incidentally on radiographs [28,29,30]. In the cases of periapical cemental dysplasia, clinical follow-up is usually sufficient, as the lesions are asymptomatic and do not require treatment, and the vitality of the associated teeth can be assessed through vitality testing. Consequently, periapical cemental dysplasia is believed to be less commonly identified in histopathologic examination results.

The findings of this study demonstrate that the most common oral pathologies are lesions caused by exposure to irritational stimuli. In order to prevent the formation of such lesions at an early stage, these stimuli should be identified and prevented at an early stage. Factors that may cause intraoral irritation, such as devitalized teeth, hot stimuli, and cheek and lip biting should also be prevented. Clinicians should be aware of these etiologic factors during routine clinical examinations.

One limitation of this study is the variation in clinicians’ approaches when selecting samples for histopathological examination, as biopsies are subject to physician discretion. This variation in clinical judgment may influence the reported prevalence of pathologies. Another limitation is that the study was conducted at a single center, which may restrict the generalizability of the findings. Additionally, as the study was retrospective, certain demographic factors—such as socioeconomic status, geographic location, occupation, and oral habits, which are important for identifying potential risk groups—could not be evaluated during the review of patient data. Despite these limitations, the results provide a comprehensive understanding of the prevalence of potential pathologies within the population. This study is expected to contribute to the literature as the only one in recent years to evaluate the prevalence data of intraoral benign pathologies without any subgroup or age restrictions. Understanding the prevalence of these lesions will aid in making more accurate clinical pre-diagnoses, planning treatment strategies, evaluating pathology distributions, and guiding future research. The findings on the frequency of pathological lesions are valuable contributions to both clinical practice and research.

## 5. Conclusions

The most common hard tissue lesion is the radicular cyst, which occurs across a wide age range and in similar proportions in both men and women. Among soft tissue lesions, traumatic fibroma is the most common, especially in women, and has been seen across a wide age range. Both conditions are typically associated with inflammation and irritation. Increasing the awareness of patients and healthcare professionals of the possible causative agents of these lesions is essential for the implementation of preventive and protective measures. The findings of this study are important for the advancement of preventive treatment approaches, as they may help to reduce these conditions before they occur.

As a result of the evaluations, it can be seen that the age range of the lesions is concentrated between the third and sixth decades of life. In terms of gender distribution, soft tissue lesions were twice as common in women compared to men. Intraosseous lesions were more common in men, although there was no significant difference. Knowing the demographic distribution and prevalence of intraoral lesions, such as age and gender, is important for identifying the target populations of lesions and increasing clinical awareness and early diagnosis. These data are necessary to facilitate clinical diagnosis and improve diagnostic accuracy. In addition, it is imperative that clinicians do not overlook rare lesions; maintaining a high diagnostic awareness of these atypical cases is critical for effective clinical management. Studies with larger sample sizes are warranted to elucidate underlying etiologic factors and the impact of regional and demographic variables on these pathologies.

## Figures and Tables

**Table 1:** The demographic distribution and frequency rates of soft tissue lesions.

Soft Tissue Lesions	Number of Patients (*n* = 75)	Prevalence Rate (%)	Female–Male Patient Count (50 Female/25 Male)	Female (F) and Male (M) Rate (%)	Age Range	Mean Age
Traumatic Fibroma	19	25.33	11/8	57.89 F 42.11 M	12–62	47.44
Fibroepithelial Hyperplasia	14	18.66	8/6	57.14 F 42.86 M	14–81	45.92
Peripheral Giant Cell Granuloma	14	18.66	12/2	85.71 F 14.29 M	8–75	49.53
Peripheral Ossifying Fibroma	8	10.66	6/2	75.00 F 25.00 M	13–51	35.00
Lichen Planus	8	10.66	6/2	75.00 F 25.00 M	40–69	55.12
Pyogenic Granuloma	4	5.33	3/1	75.00 F 25.00 M	21–56	43.50
Mucous Retention Cyst	2	2.66	2/0	100 F 0.00 M	9–70	39.50
Epidermoid Cyst	2	2.66	0/2	0.00 F 100 M	48–49	48.50
Verruca Vulgaris	1	1.33	0/1	0.00 F 100 M	47	47.00
Lupus Erythematosus	1	1.33	1/0	100 F 0.00 M	21	21.00
Amalgam Tattoo	1	1.33	1/0	100 F 0.00 M	46	46.00
Lipoma	1	1.33	0/1	0.00 F 100 M	64	64.00

**Table 2:** The demographic distribution and frequency rates of intraosseous lesions.

Intraosseous Lesions	Number of Patients (*n* = 160)	Prevalence Rate (%)	Female–Male Patient Count (73 Female/87 Male)	Female (F) and Male (M) Rate (%)	Age Range	Mean Age
Radicular Cyst	69	43.12	30/39	43.47 F 56.53 M	8–80	43.33
Periapical Granuloma	36	22.50	25/11	69.44 F 30.56 M	18–74	41.05
Dentigerous Cyst	26	16.25	4/22	15.38 F 84.62 M	6–74	31.84
Keratocystic Odontogenic Tumor	12	7.50	5/7	41.66 F 48.34 M	9–90	32.33
Incisive Canal Cyst	8	5.00	3/5	37.50 F 62.50 M	20–63	49.00
Odontoma	5	3.12	3/2	60.00 F 40.00 M	4–17	10.20
Condensing Osteitis	2	1.25	2/0	100 F 0.00 M	54–57	55.50
Periapical Cemental Dysplasia	1	0.62	1/0	100 F 0.00 M	58	58.00
Residual Cyst	1	0.62	0/1	0.00 F 100 M	74	74.00

## Data Availability

The data presented in this study are available upon request from the corresponding author. The data are not publicly available due to hospital policies.
